# Exploring the Psychosocial Impact on Families Caring for Children with Cerebral Palsy: A Qualitative Study in Saudi Arabia

**DOI:** 10.3390/healthcare14091252

**Published:** 2026-05-06

**Authors:** Norah G. Alkhaledi, Regie B. Tumala, Abdualrahman S. Alshehry, Naif H. Alanazi

**Affiliations:** College of Nursing, King Saud University, Riyadh 12372, Saudi Arabia; abdalshehri@ksu.edu.sa (A.S.A.); nalanazz@ksu.edu.sa (N.H.A.)

**Keywords:** caregiver burden, carers, children, cerebral palsy, qualitative study

## Abstract

**Background:** Parents of children with cerebral palsy (CP) experience several challenges in providing care, which can impact the child’s quality of life. CP is one of the most common neurological conditions that demand a great deal of time and effort from caregivers. **Purpose:** To explore the psychological and social effects experienced by Saudi families while rearing children with CP. **Methods:** This research utilizes qualitative research methods. The purposive sampling method was used to select 13 caregivers of children with CP from the Children with Disability Association in Riyadh, Saudi Arabia. Personal interviews were conducted with a group of parents, then these interviews were analyzed to derive the experiences of the participants. **Results:** The findings highlighted the difficulties that these families go through from the moment the diagnosis was made, as well as other care burdens, including costs, isolation, and continuous anxiety and worries about the future of the children. **Conclusions:** This study identifies the need for organized psychological and social support, which has a significant impact on enhancing adaptability and the lives of the children.

## 1. Introduction

Cerebral palsy (CP) is the most common cause of childhood physical disability. Globally, the estimated prevalence of CP is more than 17 million people, with a rate of approximately 1.5–4 per 1000 live births [1]. The incidence rate of CP varies between 2 and 3 per 1000 live births in the USA, Europe, and Australia [2] and can reach 10 per 1000 live births in Sub-Saharan Africa [3].

In Saudi Arabia, CP prevalence is estimated at approximately 2.34 per 1000 children, with growing recognition of its impact on family well-being [4]. A higher prevalence of CP-related risk factors, such as prenatal and postnatal infections and nutritional deficits, are frequently blamed for this increased incidence [5].

Families with children who have CP must deal with ongoing challenges. This illness has an impact on a child’s posture and mobility, and it frequently causes cognitive or communicative difficulties. Care demands include daily help, medical care, and rehabilitation, which puts strain on parents and the family. Research demonstrates that, aside from the child’s results, providing care affects the family’s health [6].

Research from several contexts highlights issues such as mental distress, isolation in society, and economic burden. Caregivers experience anxiety, depressive symptoms, limited social engagement, and less income resulting from decreased work hours or job losses. Expenses for therapy, equipment, and travel are also a concern. Reviews discuss unmet informational, psychological, social, and financial needs, and advocate for organized assistance such as education, counseling, peer groups, and respite programs [6,7].

Arab countries continue to produce little research on families of children with CP, and new assessments demand evidence and solutions tailored to the culture. Stress among caregivers, stigma, and gaps in service channels are highlighted in the literature. According to research from Egypt and regional evaluations, health system obstacles such as lengthy wait times and a lack of commitment to suggested measures exacerbate family stress [8,9].

Families report significant emotional burdens and practical obstacles in managing fragmented support systems. According to recent data from Saudi Arabia, families and children with CP do not receive enough psychological therapy. Nursing plays a pivotal role in assessment, caregiver education, service coordination, and advocacy for culturally appropriate psychological support [7,10].

## 2. Materials and Methods

### 2.1. Research Design

A qualitative descriptive approach was used to explore caregivers’ experiences of raising children with CP. Specifically, this study explored the psychological and social effects experienced by Saudi families raising children with CP.

### 2.2. Sampling Process

The study population consisted of primary caregivers of children diagnosed with CP. A purposive sampling strategy was used to recruit participants who could provide rich, firsthand accounts of the caregiving experience. The study’s inclusion criteria were as follows: adult caregivers (aged 18 years or older), primary responsibility for the daily care of a child with CP, and a minimum caregiving duration of one year to ensure depth of experience. The exclusion criterion is that the caregivers should not have any chronic physical or mental illnesses. Sampling continued until thematic saturation was reached, defined as the point at which no new codes or themes emerged during analysis. Saturation was initially observed after the 11th interview and confirmed through two additional interviews.

### 2.3. Setting

Data were collected within the Children with Disability Association (CDA) facility in Riyadh, Saudi Arabia. The CDA provides specialized services for children with disabilities, and offers integrated medical, educational, and rehabilitation services to promote learning, functional development, and general health. In line with the study’s objectives, this environment provided suitable access to caregivers who had personal experience of providing care for children with CP.

### 2.4. Data Collection Process

The interviews took place between November and December 2025. Participants were informed of the purposes and procedures of the study. They gave written informed consent, were made aware of their right to remain anonymous, and were free to withdraw from the study at any time.

In-depth semi-structured data collection was performed in Arabic and later transcribed and translated into English. Accordingly, interviews were based on a predetermined set of questions that were repeatedly changed to incorporate other topics as contributions permit. These questions include “Can you describe your experiences of raising a child with cerebral palsy?”, “What difficulties have you faced in this situation?” “How do these challenges affect you and your family?” and “What types of assistance do parents need when caring for a child with cerebral palsy?”.

In this way, emergent themes were incorporated into the interview procedure during data collection to ensure that the study was adequately representative of nuanced and dynamic dimensions of participants’ experiences [11].

Before starting the interview, the first author discussed the objectives and questions of the interview with the recruited participants (2 participants) to ensure that the questions are clear, unbiased, and capable of eliciting rich, meaningful data. Furthermore, the findings of the pre-testing were not included in the main study to identify any practical or logistical challenges in data collection (e.g., timing, recording, participant engagement).

Participation in this study was completely voluntary. Each participant completed a separate interview to protect the confidentiality and privacy of the information they submitted. Adherence to ethical research standards was crucial throughout the investigation. The Declaration of Helsinki [12] was followed in addressing all ethical issues. Interviews were conducted by the first author, a trained nurse researcher with experience in qualitative interviewing, and 30–35 min were allotted for the interviews. The responses of the participants were recorded using audio device. Probing questions based on the participants’ opinions were used throughout the interview to extract crucial information, such as “Why?”, “How?” and “What do you mean by this?”.

For the sake of reflexivity, the authors are not associated with the CDA. Throughout the study, the authors consistently analyzed and discussed perspectives and opinions, acknowledging that thinking might be influenced by preconceptions, prejudices, and beliefs. The authors’ keen awareness of the research roles informed their actions and choices. They minimized bias in the conclusions by performing intersubjective checks, using a reflexive strategy, and staying grounded in the facts to maintain rigor.

### 2.5. Data Analysis

Data analysis was conducted using MAXQDA 2024 (VERBI Company; Berlin, Germany). Every interview was accurately transcribed to guarantee accuracy. The data analysis process followed Braun and Clarke’s [13] six recursive steps. Several readings and the recording of initial insights are necessary to become familiar with the material. Initial coding was conducted by the first author through repeated reading of transcripts to identify meaningful segments of text. Codes were grouped into preliminary categories and reviewed by two qualitative research experts to ensure analytic rigor. Through iterative discussions, categories were refined into broader themes that reflected shared caregiver experiences.

## 3. Results

The findings are reported in accordance with the Consolidated Criteria for Reporting Qualitative Research (COREQ) guidelines [14].

Table 1 displays the socioeconomic and demographic information of the 13 participants, who are parents of children with CP. The parents range in age from 27 to 65 years, with 10 out of 13 being female. The participants’ educational levels range from no formal education to university graduate, and nine out of 13 of them are unemployed. These households have somewhere between three and seven family members. The age range of children with CP is 11 months to 10 years, and their gender distribution is equal between males and females. Seven out of 13 participants said they had enough money. One person said they did not have enough income, while five said they just occasionally had enough.

Two overarching main themes were identified through a detailed analysis of the transcripts. Themes and the sub-themes are summarized in Table 2.

### 3.1. Confronting Initial Challenges Associated with Raising a Child with CP

The journey of parents adapting to raising a child with CP is highlighted by this key theme, ‘Confronting initial challenges associated with raising a child with CP,’ which also emphasizes the particular difficulties they faced in early years. Parents reported key challenges such as identifying variations and establishing a diagnosis, challenges in accessing information, and addressing negative attitudes from family members.

#### 3.1.1. Identifying Variations and Delaying a Diagnosis

Within the context of this subtheme, the parents discussed the difficulties they faced in obtaining an accurate diagnosis for their children who were diagnosed with CP. They had to deal with extended periods of confusion and worry when an accurate diagnosis was delayed, and they frequently requested advice from several physicians to obtain an accurate answer about their child’s illness.

As evidence of this point, the following statements are presented:

*“If we had started treatment earlier, he would’ve improved more, but unfortunately, no one guided us at that time.”*

*(Participant #4)*

*“We were delayed a lot because of appointments and waiting lists, and every delay affects his condition.”*

*(Participant #9)*

*“Prior to receiving an explanation from a private physician, I was completely unaware of what cerebral palsy was.”*

*(Participant #5)*

*“After the first appointment, we waited six months for a follow-up visit.”*

*(Participant #9)*

#### 3.1.2. Barriers to Accessing Care and Support Services

The parents faced significant difficulties in obtaining information regarding services that can meet both their child’s needs and their own support requirements, representing one of the most substantial challenges during the initial years. In the absence of official resources, numerous parents relied on personal and informal networks, exerting considerable effort to collect essential information. This situation frequently resulted in fatigue and a high level of tension. The following quotes illustrate this matter:

*“I had no idea where to go or what to do. I was uncertain regarding potential resources or the appropriate therapeutic interventions for my child. Initially, I experienced a sense of being trapped and overwhelmed.”*

*(Participant #1)*

*“I was independently searching for the facility. I traveled from another city due to the insufficient number of physical therapists in our local area. The treatment facility is located three hours away, and we commute there twice weekly.”*

*(Participant #13)*

*“I was trying to search on social media for people who have children with the same condition as my son to find out how and where to start treatment.”*

*(Participant #7)*

#### 3.1.3. Addressing Negative Attitudes from Family Members

Some parents reported being highly stressed because of the adverse reactions of their relatives toward their child with a disability. They reported that they stopped participating in social engagements due to concerns regarding potential judgment, expressions of pity, or disparaging comments from others, subsequently resulting in feelings of humiliation and unease. This situation is demonstrated by the following quotations:

*“I stopped going out because people look at him curiously or with sympathy.”*

*(Participant #10)*

*“I don’t like being in public because I always feel like I need to describe my child’s condition.”*

*(Participant #2)*

*“Even some relatives started to distance themselves—as if having a child like this is a shame.”*

*(Participant #3)*

### 3.2. Overcoming the Difficulties of Everyday Family Life While Caring for a Child with CP

This theme addresses the challenges encountered by parents in managing daily family life with a child diagnosed with CP. Regardless of individual circumstances, parents frequently reported financial strain, insufficient social support when providing care, and emotional strain from concerns and unfulfilled requirements.

#### 3.2.1. Financial Strain

Parents are perpetually burdened by financial difficulties, and they are concerned about their inability to finance their children’s treatment expenses. Financial strains and difficulties disrupt attention, cause insecurity and stress, adversely impact mental wellness, diminish the capacity to handle daily obstacles, and impose a significant psychological burden.

*“It’s too expensive for me to keep going to private therapy.”*

*(Participant #11)*

*“There is government help, but the services are slow and limited.”*

*(Participant #4)*

*“I would put him in a better rehab center if I had more money.”*

*(Participant #2)*

#### 3.2.2. Social Isolation and Societal Stigma

Concerns about judgment, pity, or disparaging comments from others have led caregivers to withdraw from social activities. The child’s obvious impairment frequently attracts unwelcome attention, which makes them feel uncomfortable and embarrassed, as evidenced by the following quotations:

*“When I go out, people look at him with sympathy or curiosity.”*

*(Participant #6)*

*“Sometimes, I feel like I have to describe my child’s condition in public, which makes me uncomfortable.”*

*(Participant #12)*

*“Some family members even started to stay away, as if having a child like this were a bad thing.”*

*(Participant #4)*

#### 3.2.3. Considering the Future of the Child

This subtheme illustrates the fear and confusion that parents feel while considering the future of their child with CP. It also reflects their hopes and anxieties as well as the significant responsibility they bear for their child’s care going forward.

*“Will he find a place in society? I often wonder. Is he ever going to be self-sufficient?”*

*(Participant #2)*

*“My heart hurts from worrying about his future, even as I work to train and prepare him.”*

*(Participant #13)*

## 4. Discussion

This paper examines the psychological and social effects experienced by families raising children with CP, and the findings evidently show that these families have suffered significantly not only from the point of diagnosis but also from dealing with their lives on a day-by-day basis. The participants’ experiences illustrate that delayed diagnosis and lack of clear information are among the factors that contribute most to the increased anxiety and stress they felt during the early years of their child’s life [15]. Shevell and Shevell [16] reviewed common findings from studies conducted in 1990 [17], 2000 [18], and 2010 [19] regarding parents’ perceptions of delays in disclosing the diagnosis of CP. Families still complain about delays in CP diagnosis despite several efforts to reduce them [20]. According to Baird et al. [18], parents of infants who were diagnosed later consistently expressed more dissatisfaction, saying that they would have preferred to have been informed of the potential consequences of their child’s being preterm.

The results also showed that economic burdens are a continuous struggle for families because of the high costs of physical therapy sessions, assistive devices, and transportation to rehabilitation centers, along with the loss of job opportunities or reduced working hours of some parents. The findings justify previous studies showing how economic burdens affect the mental health of caregivers, impairing their ability to care for their children effectively [15].

Thus, this study supports earlier findings that social marginalization and institutional inadequacies, in addition to the burden of care, cause emotional distress in parents [21]. Initially, there was unity in society, according to a similar study conducted in Goa with 12 parents. Social conflicts and stigmatization developed over time, eventually resulting in solitude. Parents allegedly faced more social difficulties and had trouble adjusting [22]. Parents’ helplessness is further compounded by fragmented service delivery and a lack of awareness. This disparity emphasizes the urgent need for formal psychosocial training modules to teach parents about disabilities, coping mechanisms, and advocacy tactics. According to Yue et al. [23] and Riquelme et al. [24], improved child outcomes are closely correlated with increased parental efficacy and self-competence.

Our participants reported negative attitudes from family members. This finding is in line with a study by Yoder et al. [25], where caregivers faced several behaviors, including labeling, avoidance, and abusive language, and thus used spiritual reframing as a coping mechanism.

The experiences reported by parents in this study show notable similarities with those described in the literature on other childhood disabilities, particularly autism spectrum disorder (ASD). Consistent with previous research, parents commonly report emotional strain, uncertainty about the future, and challenges related to social participation and stigma [26,27]. These shared experiences suggest that many aspects of caregiver burden are not condition-specific but rather reflect the broader demands of raising a child with a disability. However, some differences are evident. For example, parents of children with CP often emphasize the physical demands of caregiving and the need for ongoing medical and rehabilitation services. By contrast, parents of children with ASD more frequently report challenges related to behavioral management and communication difficulties [28]. This comparison highlights both the common psychosocial impact across disabilities and the challenges associated with specific conditions, reinforcing the importance of support interventions.

Raising a child with CP in Saudi Arabia involves complex challenges that reflect global patterns and culturally specific experiences. In line with global research, caregivers report high levels of stress, anxiety, and diminished quality of life, primarily due to the responsibilities of caregiving and fear regarding the child’s future [29,30]. Some studies reported similar findings in both high- and low-income countries, where emotional strain and caregiver burden remain common [31,32]. These issues are not only common within the context of Saudi Arabia but are also observed worldwide, as reported by various reviews highlighting concerns regarding mental health and substance abuse among parents [33], increased caregiver burden linked to depressive symptoms and diminished quality of life [34], need for family-centered care pathways [35], and interventions aimed at enhancing the psychological well-being of caregivers raising children with CP [36]. The Saudi context adds important layers to this experience. Family life is often grounded in strong social ties, and support from extended family members can ease some of the daily pressures associated with caregiving. Regional studies highlight how this sense of shared responsibility can provide emotional and practical relief [37].

These findings emphasize the necessity of situating caregiver experiences within their cultural context while acknowledging the universal challenges associated with raising a child with CP.

### 4.1. Limitations

In spite of the important results of this study, some determinants might affect the interpretation and generalization of the results. The study involved a small population of participants, which may limit the generalizability of the findings to all families that may be taking care of children with cerebral motor disability. Furthermore, data collection was conducted via personal interviews, which may introduce biases into the results and make them subjective in nature.

### 4.2. Implications for Clinical Practice

The results indicate the need for practical and continuous assistance for families with children with cerebral motor disabilities, highlighting significant psychological, social, and financial burdens that parents have to bear. Health services must be responsible for educating and enlightening the parents about the nature of disability and the methods of care, such as teaching them home rehabilitation strategies and approaches. In addition, psychological and social support should be extended on a continuous basis, through individual or group counseling, the creation of support groups where parents can share experiences, and programs contributing to a reduction in social isolation and stigma, with the goal of enhancing parents’ feelings of competence and control over their everyday life. Healthcare professionals and policymakers need to ensure that caregivers can access occupational and physiotherapy through strong financial support programs, in addition to caregiver support programs, parent education and training initiatives, early diagnostic pathways, integrated rehabilitation services, and psychosocial support within pediatric care systems.

Implementing these recommendations in practice could significantly contribute to alleviating families’ daily pressures, improving their psychological and social adjustment, and the overall quality of life for children and their families. This approach can also help enhance families’ participation in rehabilitation programs and achieve better long-term outcomes for both the child and the family.

## 5. Conclusions

This study indicates that childcare in the presence of motor impairment poses a substantial challenge to families, as psychosocial and economic issues interact and affect the management of the lives of the child and the family. A lack of diagnosis and access to information and services, financial distress, a lack of socialization due to association with disability, and fear for the future and autonomy of the child are additional concerns related to the responsibility of taking care of the child. All these factors affect the mental health and emotions of parents.

The findings also underscore the important active role of health teams, particularly nurses, social workers, and physical therapists, in providing support to families, coordinating services, monitoring child development, and ensuring that interventions are culturally appropriate and meet the needs of each family. Adopting this integrated approach can reduce daily stress, improve family psychosocial adjustment, and enhance the child’s long-term well-being.

Ultimately, the research indicates that improving the lives of children with CP depends largely on supporting the entire family, providing appropriate services, and enhancing community participation, which helps build a more supportive and inclusive environment for the child and family, and achieves sustainable positive results at the psychological and social levels.

## Figures and Tables

**Table 1:** Socio-demographic profiles of the caregivers (*n* = 13).

Participant	Age of Parents	Sex of Parents	Educational Level	Employment Status	Number of Family Members	Age of Child with CP	Sex of Child with CP	Enough Income
P1	36	Female	Diploma	Unemployed	5	3 Years	Male	Yes
P2	30	Female	University	Unemployed	4	2 Years	Female	Yes
P3	48	Male	High School	Employee	7	7 Years	Female	Sometime
P4	32	Female	University	Unemployed	4	11 Months	Female	Sometimes
P5	65	Male	Primary School	Unemployed	3	3 Years	Male	Sometimes
P6	27	Female	High School	Unemployed	3	5 Years	Female	Yes
P7	36	Female	University	Unemployed	5	3 Years	Male	No
P8	46	Male	High School	Employee	5	7 Years	Male	Yes
P9	34	Female	Middle School	Unemployed	7	10 Years	Male	Yes
P10	38	Male	High School	Unemployed	5	9 Years	Female	Sometimes
P11	32	Female	No Formal Education	Unemployed	7	9 Years	Male	Yes
P12	29	Female	High School	Unemployed	5	8 Years	Female	Yes
P13	31	Female	Middle School	Unemployed	5	4 Years	Male	Sometimes

*Note.* CP = Cerebral palsy.

**Table 2:** Thematic analysis results.

Theme	Sub-Theme
Confronting initial challenges associated with raising a child with CP	▪ Identifying variations and delaying a diagnosis ▪ Barriers to accessing care and support services ▪ Addressing negative attitudes from family members
Overcoming the difficulties of everyday family life while caring for a child with CP	Financial strain Social isolation and societal stigma Considering the future of the child

*Note.* CP = Cerebral palsy.

## Data Availability

The original contributions presented in this study are included in the article. Further inquiries can be directed to the corresponding authors due to ethical restrictions. The data are not publicly available due to privacy or ethical restrictions.

## Abbreviations

The following abbreviations are used in this manuscript:

CP	Cerebral Palsy
KSA	Kingdom of Saudi Arabia
CDA	Children with Disability Association
